# Antisense Oligonucleotide Rescue of Deep-Intronic Variants Activating Pseudoexons in the 6-Pyruvoyl-Tetrahydropterin Synthase Gene

**DOI:** 10.1089/nat.2021.0066

**Published:** 2022-10-14

**Authors:** Ainhoa Martínez-Pizarro, Fátima Leal, Lise Lolle Holm, Thomas K. Doktor, Ulrika S.S. Petersen, María Bueno, Beat Thöny, Belén Pérez, Brage S. Andresen, Lourdes R. Desviat

**Affiliations:** ^1^ Centro de Biología Molecular Severo Ochoa UAM-CSIC, Centro de Diagnóstico de Enfermedades Moleculares (CEDEM), CIBERER, IdiPaz, Universidad Autónoma de Madrid, Madrid, Spain.; ^2^ Department of Biochemistry and Molecular Biology, University of Southern Denmark, Odense, Denmark.; ^3^ Congenital Metabolic Diseases Unit, Hospital Virgen del Rocio, Sevilla, Spain.; ^4^ Division of Metabolism, University Children's Hospital Zürich, Zürich, Switzerland.

**Keywords:** pseudoexons, splicing, antisense oligonucleotides, tetrahydrobiopterin

## Abstract

We report two new 6-pyruvoyl-tetrahydropterin synthase splicing variants identified through genomic sequencing and transcript analysis in a patient with tetrahydrobiopterin deficiency, presenting with hyperphenylalaninemia and monoamine neurotransmitter deficiency. Variant c.243 + 3A>G causes exon 4 skipping. The deep-intronic c.164–672C>T variant creates a potential 5′ splice site that leads to the inclusion of four overlapping pseudoexons, corresponding to exonizations of an antisense short interspersed nuclear element *AluSq* repeat sequence. Two of the identified pseudoexons have been reported previously, activated by different deep-intronic variants, and were also detected at residual levels in control cells. Interestingly, the predominant pseudoexon is nearly identical to a disease causing activated pseudoexon in the *F8* gene, with the same 3′ and 5′ splice sites. Splice switching antisense oligonucleotides (SSOs) were designed to hybridize with splice sites and/or predicted binding sites for regulatory splice factors. Different SSOs corrected the aberrant pseudoexon inclusion, both in minigenes and in fibroblasts from patients carrying the new variant c.164–672C>T or the previously described c.164–716A>T. With SSO treatment PTPS protein was recovered, illustrating the therapeutic potential of the approach, for patients with different pseudoexon activating variants in the region. In addition, the natural presence of pseudoexons in the wild type context suggests the possibility of applying the antisense strategy in patients with hypomorphic *PTS* variants with the purpose of upregulating their expression to increase overall protein and activity.


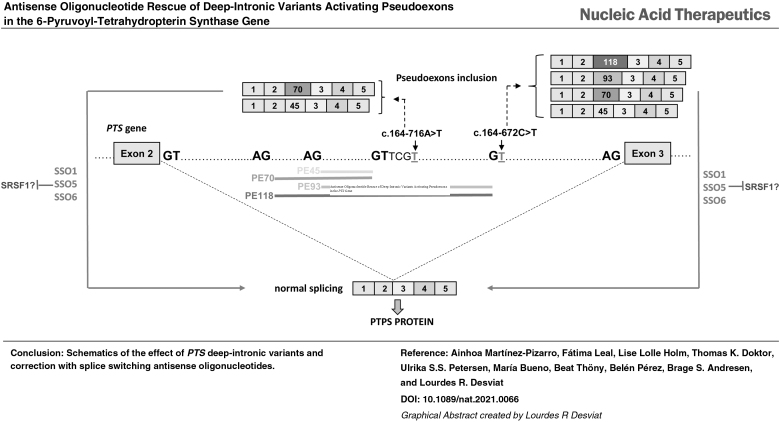


## Introduction

The PTPS (6-pyruvoyltetrahydropterin synthase; EC 4.2.3.12) enzyme, encoded by the *PTS* gene (OMIM *612719), catalyzes the second step in tetrahydrobiopterin (BH_4_) synthesis from GTP in humans. BH_4_ is the natural cofactor for phenylalanine, tyrosine and tryptophan hydroxylases, nitric oxide synthases, and glycerol ether monooxygenase [1]. PTPS deficient patients present with hyperphenylalaninemia, and most of them present severe neurological manifestations, including truncal hypotonia, bradykinesia, oculogyric crises, hyperthermia, seizures, and impaired neurophysiological development. A subset of patients exhibits the less common atypical/peripheral form with minor or no changes in neurotransmitter levels. Treatment is based on oral BH_4_ administration and supplementation with L-Dopa and 5-hydroxytryptophan, the precursors for catecholamines and serotonin, respectively, to normalize monoamine neurotransmitters in the central nervous system [2]. Despite treatment, many patients have complications with neurological and developmental problems [3].

In this work we describe a novel deep intronic variant affecting splicing in the *PTS* gene. It is well known that splice defects significantly contribute to human disease [4,5]. Splice altering variants occur beyond disruption of the canonical splice sequences at exon-intron junctions [consensus 3′ and 5′ splice sites (ss), polypyrimidine tract, and branch point], which are easily identified. Intronic and exonic splice silencers and enhancers (ISSs, ESSs, ISEs, and ESEs, respectively), bound by regulatory splice factors, are less well conserved, and sequence variants that disrupt them can be misclassified as silent or missense, unless transcript analysis is performed, which is not usual in a diagnostic setting [6,7]. In addition, aberrant transcripts can result from deep intronic variants activating or creating potential splice sites, which are used in combination with other cryptic splice sites nearby resulting in the exonization of intronic regions known as pseudoexons [8,9].

Pseudoexons share characteristics marking them as high-risk sites in the human genome, where single nucleotide variants (SNVs) may cause disease by splicing activation [10,11]. Again, these splice defects and the responsible variants are not detected in routine genetic analysis focusing in exome sequencing. Recent studies have revealed the potential of high-throughput transcriptome analysis for increasing the diagnostic rate in heterogeneous Mendelian disorders, allowing identification of splice-altering variants in both exonic and deep-intronic regions [12–16].

RNA targeted therapies such as splice switching antisense oligonucleotides (SSOs) provide an avenue toward therapy for specific splicing defects [17]. SSOs are designed to modulate splicing by sterically blocking splice sites or splicing regulatory sequences in the pre-mRNA. Blocking of consensus splice sites or splice enhancers will result in exon skipping, a strategy that has been used to bypass a mutated exon and/or to restore the open reading frame disrupted through genomic deletions, as is the case for Duchenne muscular dystrophy (DMD) [17]. In contrast, blocking splice silencers may instead promote exon inclusion, which is the therapeutic strategy used in spinal muscular atrophy (SMA) [18]. Therapeutic SSOs for DMD and SMA have been approved by the Food and Drug Administration (FDA)/European Medicines Agency (EMA), and several others are in clinical trials [19,20].

SSOs have also been used to prevent aberrant pseudoexon inclusion in the mRNA. Many pseudoexons are transposable sequence elements derived from short or long interspersed nuclear elements (SINEs or LINEs, respectively) [21], and their activation by point mutations has been described as pathogenic mechanism in many human disorders [8,22–24]. In neuromuscular diseases, deep intronic variants have been identified in 77 different genes [14]. In inherited retinal diseases, therapeutic potential of SSOs have been shown for deep-intronic pseudoexon activating variants in the *CEP290*, *USH2A*, *OPA1*, and *ABCA4* genes, with an ongoing phase 1/2 clinical trial using SSOs to treat *CEP290*-associated Leber congenital amaurosis (NCT03140969) [23]. Recently, an SSO (Milasen) targeting an activated pseudoexon identified in a single patient with Batten's disease was developed and approved by FDA for clinical testing within 1 year, representing an extreme example of the use of SSOs as personalized medicines [25].

Previous studies by us and other groups reported the pathological activation by two different variants of overlapping pseudoexons in intron 2 of the *PTS* gene, which could be effectively reverted by use of a specific SSO [26]. We now report a novel deep intronic variant in the same region creating a 5′ss and activating the same pseudoexons plus two others that use the new splice site. The patient carries on the other allele an also novel variant affecting the 5′ss of exon 4. We have used transcript profiling in minigenes and in patients' fibroblasts to dissect the pathogenic mechanism for each variant and designed and tested a series of SSOs to block the insertion of the activated pseudoexons in the mature mRNA. Our results confirm SSO mediated pseudoexon skipping as a potential therapeutic approach for PTPS deficiency and point to the identification of high risk sites for the activation of *Alu-*derived pseudoexons.

## Materials and Methods

### Patient samples and genetic and biochemical analysis

Patient 1 was referred to the laboratory for biochemical and genetic analysis after giving a positive result in the neonatal screening for hyperphenylalaninemia. Biochemical analysis (pterin levels in blood and urine, and monoamine neurotransmitter levels in cerebrospinal fluid (CSF), measured by standard procedures [27]) indicated a clinical suspicion of PTPS deficiency. Genetic analysis was performed in DNA isolated from blood samples, by use of Massive Parallel Sequencing using the Clinical-Exome Sequencing TruSight™ One Gene Panel (Illumina) and filtering for variants in all genes known to be responsible for hyperphenylalaninemia (*GCH1, PTS, PCBD1, QDPR, DNAJC12,* and *PAH*). *PTS* transcript analysis was performed in RNA extracted from fibroblast samples. Potentially pathogenic variants were confirmed by Sanger sequencing. Patient 2 has been previously reported [26]. Ethical approval for the present study was granted by the Institutional Ethics Committee (Universidad Autónoma de Madrid).

### Fibroblasts and cell lines

Patients' fibroblasts were referred to the laboratory for genetic analysis. Control fibroblasts CC2509 were obtained from Lonza. They were cultivated according to standard procedures and used with number of passes below 20. Briefly, cells were maintained in Minimum Essential Medium (MEM; Sigma Aldrich) supplemented with 1% (v/v) glutamine, 10% fetal bovine serum (FBS; Gibco), and 0.1% antibiotic mix (penicillin/streptomycin) under standard cell culture conditions [37°C, 95% relative humidity, 5% carbon dioxide (CO_2_)]. Cells were treated with puromycin (200 μg/mL) 5 h before harvesting for transcript analysis, to inhibit nonsense mediated mRNA decay (NMD).

For minigene analysis, human hepatoma cells Hep3B and SH-SY5Y neuroblastoma cells were used. Cells were grown in MEM for Hep3B and Dulbecco's modified Eagle's medium (DMEM; Gibco) for SH-SY5Y, both supplemented with 5% FBS, 1% glutamine, and 0.1% antibiotic mix under standard cell culture conditions (37°C, 95% relative humidity, 5% CO_2_).

### Minigenes

For *in vitro* evaluation of the splicing profile different minigene constructs were used. The previously described splicing reporter plasmid encompassing *PTS* exons 2–4 cloned in the pCR3.1 vector [26,28] was used as template for deep intron 2 variants c.164-672C>T and c.164-716A>T. The pSPL3 vector (Life Technologies) was used in the analysis of the c.243 + 3A>G variant. Using this vector, we cloned *PTS* exon 4 and ∼150 bp of the flanking intronic regions, amplified from genomic DNA using specific primers (Forward: 5′-GCTTCCATGCTGAGGTCAAT-3′ and Reverse: 5′-ACTATTCCCCAACACCCACA-3′). Gene fragments were cloned into the pGEMT vector (No. A1360; Promega). The insert was excised with *Eco*RI and subsequently cloned into pSPL3. Variant minigenes containing the desired nucleotide changes were generated by site-directed mutagenesis with QuikChange Lightning Kit (Agilent Technologies, Santa Clara, CA, USA) using primers introducing the change and their reverse complement. Sanger sequencing confirmed the identity of the constructs.

### SSO design

The sequence of the region encompassing the pseudoexons was analyzed and SSOs designed following published guidelines [29,30]. Splice enhancer motifs were determined using ESE finder3.0. Pre-mRNA secondary structure was predicted using m-fold (www.unafold.org/mfold/applications/rna-folding-form.php), and RNAstructure software (https://rna.urmc.rochester.edu/RNAstructureWeb/) was used to predict secondary structure resulting from the interaction antisense oligonucleotide (AON)-target RNA. Specificity of the selected SSOs was verified using BLAST (https://blast.ncbi.nlm.nih.gov/Blast.cgi). SSOs were 20- or 25-mers with a Tm above 60°C and a GC content between 50% and 60%. The exact sequences (Supplementary Table S1) and their location are depicted in Fig. 3A. A scrambled SSO was used as negative control. Given the abundance of *Alu* elements in the genome, we evaluated closely the predicted off-target binding sites, confirming that they are far from adjacent exons. All oligonucleotides were synthesized as full 2′-OMe phosphorothioates by Sigma or Eurogentec.

### Transfections and splicing analysis in minigenes

For minigene assays, Hep3B or SH-SY5Y cells were seeded in six-well plates at a density of 4 × 10^5^ or 8 × 10^5^, respectively, in 2 mL MEM or DMEM 5% FBS and grown overnight. Cells were transfected using Lipofectamine 2000 (Invitrogen) with 2 μg per well of the corresponding plasmid minigene. For SSO treatment, cells were cotransfected with 2 μg of wild type or mutant minigenes and 50 nM of each SSO. Cells were harvested by trypsinization 48 h after transfection. Total RNA was isolated using TRIzol Reagent (ThermoFisher) and phenol–chloroform extraction. cDNA synthesis was performed using NZY First-Strand cDNA Synthesis Kit (NZYtech).

Splicing analysis was carried out by polymerase chain reaction (PCR) amplification with FastStart Taq DNA Polymerase (Roche) using specific primers SD6 (5′- TCTGAGTCACCTGGACAACC-3′) and SA2 (5′- ATCTCAGTGGTATTTGTGAGC-3′) for pSPL3 minigenes and PL3 (5′-GGGAGACCCAAGCTGGCTA-3′) and PL4b (5′-AGTCGAGGCTGATCAGCGG-3′) for the pCR3.1 minigene. The amplification products were analyzed by 2% agarose gel electrophoresis. Pseudoexon bands were excised from the gel and purified using QIAquick Gel Extraction (Qiagen). The identity of each pseudoexon was confirmed by subcloning in pGEMT (Promega) and subsequent Sanger sequencing.

### Transfections and splicing analysis in fibroblasts

For SSO treatment, fibroblasts were seeded in six-well plates at a density of 3 × 10^5^ in 2 mL MEM 10% FBS and reverse transfected at the same time with 50 nM SSOs using Lipofectamine 2000. Cells were harvested by trypsinization after 48 h. RNA was isolated using TRIzol Reagent (ThermoFisher) and phenol–chloroform extraction. Reverse transcription PCR (RT-PCR) analysis was performed using primers *PTS* cDNA F (5′-ATGAGCACGGAAGGTGGTG-3′) and Exon 3-R (5′-CTCTCCATGTACTGTCACCACAA-3′) to amplify a fragment from exon 1 to exon 3. The amplification products were analyzed by 2.5% agarose gel electrophoresis and their identity and quantification confirmed by deep sequencing.

### Western blot analysis

Approximately 4 × 10^5^ fibroblasts were seeded per well in a six-well plate with 50 nM of each SSO, which were reverse transfected using Lipofectamine 2000. Ninety-six hours after transfection cells were harvested by trypsinization and lysed by freeze-thawing in a lysis buffer (10 mM Tris-HCl pH 7.5, 10% glycerol, 150 mM sodium chloride, and 0.1% Triton X-100) with protease inhibitor (Roche). Protein concentration was determined using Bradford reagent (Bio-Rad).

Fifteen micrograms of protein extract from control sample and 75 μg from patients' samples were electrophoresed on 12% SDS-polyacrylamide gels, transferred to a nitrocellulose membrane in an iBlot transfer device (Invitrogen) and blocked in Tris buffered saline-0.1%Tween supplemented with 5% nonfat dry milk. Membranes were incubated overnight at 4°C with the primary antibody (PTPS 1:500, sc-514628; Santa Cruz Biotechnologies) followed by a secondary horseradish peroxidase-conjugated anti-mouse IgG (1:2000, sc-514628; Cell Signaling). GAPDH was used as a loading control (using primary antibody at ab8245, 1:5000; Abcam). Proteins were detected using the Enhanced Chemiluminescence reagent (GE Healthcare). Images were scanned with a densitometer GS-900 (Bio-Rad) and quantified with Image Lab 5.2 (Bio-Rad).

### Deep sequencing

Amplicons were generated in a first PCR with fusion primers containing a specific sequence plus a common tag (**CS1** and CS2):

- Forward primer: 5′-**ACACTGACGACATGGTTCTACA**CTGTTTGGGAAATGCAACAA-3′
- Reverse primer: 5′-TACGGTAGCAGAGACTTGGTCTTCTCCATGTACTGTCACCACAA-3′

Libraries preparation and sequencing were carried out at Fundación Parque Científico de Madrid under protocols developed and optimized for next generation amplicon sequencing. Briefly, amplicons were diluted 1/50, and 1 μl was used as input for a second PCR of 10 cycles performed with a High-Fidelity DNA Polymerase in the presence of primers [5′-AATGATACGGCGACCACCGAGATCT**ACACTGACGACATGGTTCTACA**-3′ and 5′-CAAGCAGAAGACGGCATACGAGAT-(10 nucleotides barcode)-TACGGTAGCAGAGACTTGGTCT-3′] of the Access Array Barcode Library for Illumina Sequencers (Fluidigm). The finally obtained amplicons were validated and quantified by Bioanalyzer, and equimolecular pool was purified by AMPure XP beads (Beckman Coulter). This pool was titrated by quantitative PCR using the “Kapa-SYBR FAST qPCR Kit for LightCycler480” and a reference standard for quantification. Amplicon pool was denatured before being seeded on a flow cell where clusters were formed and sequenced using a “MiSeq Reagent Kit v3,” in a 2 × 300 pair-end sequencing run on a MiSeq sequencer.

### RNA pull down

Affinity purification of RNA-binding proteins was performed as previously described [31], using 3′ end biotinylated RNA oligonucleotides (LGC Biosearch Technologies, Risskov, Denmark): PTS1 (5′-CCAGGUUCGAGCGAUUCUCCAGCCU-3′), PTS1MUT1 (5′-CCAGGUUCGUGCGAUUCUCCAGCCU-3′), PTS1MUT2 (5′-CCAGGUUCUAGCUAUUCUCCAGCCU-3′), PTS1Rev (5′-AGGCUGGAGAAUCGCUCGAACCUGG-3′), PTS5 (5′-UUCUCCAGCCUCAGCCUCCCGAGUA-3′), PTS5MUT (5′-UUCUCCAGCCUCAGCCUCUUGAGUA-3′), PTS5Rev (5′-UACUCGGGAGGCUGAGGCUGGAGAA-3′), PTS6 (5′-UCCCGAGUAGCUGAGAUUACAGGUG-3′), PTS6MUT (5′-UCUUGUGUAGCUUAUAUUUCUGGUG-3′), and PTS6Rev (5′-CACCUGUAAUCUCAGCUACUCGGGA-3′).

Five hundred picomole of each biotinylated RNA oligonucleotide was immobilized in 25 μL Dynabeads M-280 Streptavidin magnetic beads (Invitrogen) and incubated with 293 cell nuclear extract (Protein One). Blank beads were added as a control for background binding. Proteins were eluted in 25 μL protein sample buffer and analyzed by western blotting with antibodies against SRSF1 (32–4500 from Invitrogen), hnRNP A1 (R9778 from Sigma Aldrich), SRSF2 (04-1550 from Millipore), and SR proteins (33–9300 from Invitrogen).

### Surface plasmon resonance imaging

Surface plasmon resonance imaging (SPRi) was carried out as previously described [32]. Briefly, the biotinylated RNA-oligonucleotides described above were immobilized onto a G-strep sensorchip (SSENS) for 20 min. The following recombinant proteins were injected for 8 min, followed by dissociation for 4 min; 25–800 nM SRSF1 (GenScript) and 6.25–200 nM hnRNPA1 (ab123212; Abcam) with a continuous flow of SPR buffer (10 mM Tris-HCl, ph 7.9, 150 mM potassium chloride, 3.4 mM ethylenediaminetetraacetic acid, 0.005% Tween 20). Nuclear extract was used as a control of the oligos binding efficiency. Binding was fitted to a 1:1 kinetics model with Scrubber2 (v. 2.1.1.0; Biologics Inc.). For hnRNPA1 a biphasic 1:2 model was used in ClampXP (version 3.50; Biosensor Data Analysis).

### *In silico* analysis

The effect of the variants on splice site strengths and the presence of putative splicing regulatory elements were predicted using Human Splice Finder (HSF) (http://www.umd.be/HSF/) [33], MaxEntScan (http://genes.mit.edu/burgelab/masent/Xmaxentscan_scoreseq.html) [34], ESEFinder 3.0 software (http://rulai.cshl.edu)[35], and RBPmap (http://rbpmap.technion.ac.il/) [36].

The presence of repetitive sequences was analyzed using RepeatMasker (http://www.repeatmasker.org/).

### Processing of cross-linking immunoprecipitation datasets

Raw SRSF1 eCLIP reads [37] were downloaded from the ENCODE website and trimmed twice using cutadapt v1.15 [38]. Raw PAR-CLIP reads were downloaded from the GEO repository using the accession number GSE71095 [39] and trimmed using cutadapt. Raw HITS-CLIP reads were downloaded from the GEO repository using the accession numbers GSE131745 [40] and trimmed using cutadapt. Subsequently, reads were mapped with STAR v2.7.8a [41] to the human genome using GENCODE v39 hg38 annotations allowing for up to 100 mapping locations. Run parameters and further details are described in Supplementary Methods S1.

### Visualization of CLIP reads

We used Gviz [42] to visualize the alignments along with GenomicFeatures [43] to parse the GENCODE GTF file with transcripts.

## Results

### Clinical, biochemical, and genetic analysis

Patient 1 was detected in newborn screening due to high Phe levels (226 μM) and later diagnosed as the severe form of PTPS deficiency based on urinary pterins (elevated neopterin, 22.9 mmol/mol creatinine and low biopterin, 0.3 mmol/mol creatinine) and monoamine neurotransmitter deficiency in CSF, corresponding to very low levels of homovanillic acid and of 5-hydroxyindoleacetic acid, (140 nM and 75 nM, respectively; for reference values see [44]). Prolactin, which is physiologically inhibited by dopamine, showed very high values (655–1590 μU/mL; reference values 127–637 μU/mL). Treatment with sapropterin (BH_4_), 5-OH Trp, and L-Dopa/carbidopa, adjusted to body weight, was implemented. Currently, the patient is 6 years old and developing normally, with no overt clinical symptoms.

Genetic analysis by exome sequencing identified a single variant in the *PTS* gene: c.243 + 3A>G, affecting the 5′ss of exon 4 (decrease in HSF splicing score from 97.71 to 81.74 and from 8.7 to 4.4 in MaxEnt score). PTPS deficiency is inherited in autosomal recessive manner, so we sought to find the second pathogenic variant, which might reside within regions not covered by routine exome sequencing. We used Sanger sequencing to analyze a candidate region in intron 2 in which two different pathogenic variants were previously shown to activate a series of overlapping pseudoexons [26,28]. This analysis revealed the presence of a novel variant c.164-672C>T that creates a potential 5′ss (HSF splicing score 75.85 and MaxEnt 5.8), downstream of the previously identified pseudoexons.

**FIG. 1.**
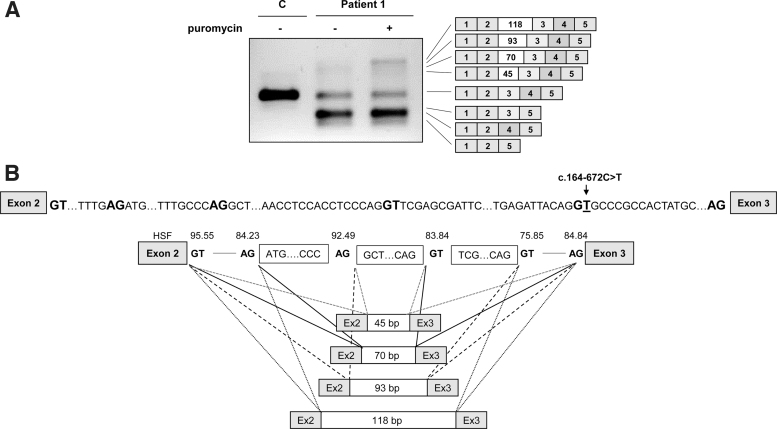
*PTS* gene splicing profile in patient 1 fibroblasts. **(A)** RT-PCR analysis in C and patient 1 fibroblasts treated with (+) or without (−) puromycin. Shown on the *right* are the schematics of the identified bands confirmed by sequencing. **(B)** Sequence of the intron 2 region where the pseudoexons are located, showing the conserved GT and AG splice sites (in bold) used by the pseudoexons and the location of the novel variant. Shown below is the schematics of the identified pseudoexons along with the score of the splice sites according to HSF. C, control; HSF, Human Splice Finder; RT-PCR, reverse transcription polymerase chain reaction.

RT-PCR analysis in patient's fibroblasts revealed several aberrant bands of higher and lower molecular weight compared to the correctly spliced band, which was detected in low amounts (Fig. 1A). The lower sized bands were prominent in samples treated or not with puromycin, used as NMD inhibitor. After subcloning and sequencing we identified transcripts with exon 3 skipping [out of frame, p.(Val55Aspfs*2), occurs naturally due to poor exon definition] [45], exon 4 skipping [in frame, p.(Ile63_Glu81del)], and skipping of both exons 3 and 4 p.(Val55Glyfs*8).

In puromycin treated samples faint high molecular weight bands were observed, which were identified by subcloning and sequencing analysis as insertion of several intronic sequences (pseudoexons) of 45, 70, 93, and 118 bp between exons 2 and 3. Pseudoexons of 45 and 70 bp (PE45 and PE70) have been previously reported to be activated by different variants in the region [26] and those of 93 and 118 bp (PE93 and PE118) correspond to the use of the newly created 5′ss by variant c.164-672C>T, in combination with upstream cryptic 3′ss (Fig. 1B). All correspond to exonizations of an antisense *AluSq* sequence, as previously described [28]. The newly created 5′ss corresponds to *Alu* position 156, present in naturally exonized *Alu*s [46,47].

Interestingly, by altering the conserved C at position 156 in the *AluY* consensus sequence the *PTS* c.164-672C>T variant alters this to a 5′ss, identical to that used in the disease associated *F8* pseudoexon [24,48]. The *PTS* PE118 is nearly identical to the *F8* pseudoexon with the same 3′ss and 5′ss, consistent with the fact that this seems to be the most prominent *PTS* PE isoform. Insertion of *PTS* PE45 and PE93 results in extra amino acid insertions (p.(Val55_Val56ins15) and p.(Val55_Val56ins31), respectively), while insertion of PE70 and PE118 results in frameshift and introduction of premature stop codons [p.(Val55Serfs*33) and p.(Val55Aspfs*37), respectively].

### Functional analysis in minigenes of the novel variants

**FIG. 2.**
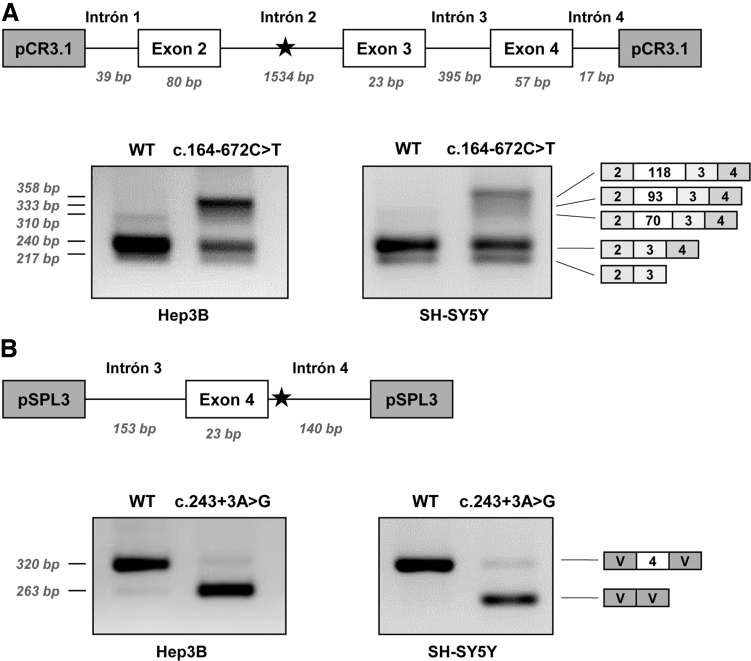
Minigene analysis of the novel splicing variants identified in patient 1. **(A)** Scheme of the pCR3.1 minigene used and RT-PCR analysis of WT and mutant (c.164–672C>T) minigenes in Hep3B and SH-SY5Y cells. **(B)** Scheme of the pSPL3 minigene used and RT-PCR analysis of WT and mutant (c.243 + 3A>G) minigenes in Hep3B and SH-SY5Y cells. The *star* indicates the location of the variants. WT, wild-type.

The individual effect on splicing of the novel variants was studied using minigenes transfected in Hep3B cells. Variant c.164-672C>T was analyzed in the previously described pCR3.1 minigene encompassing *PTS* exons 2–4 [26]. The mutant minigene produces, in addition to a normal sized, correctly spliced band, larger bands which after subcloning and sequencing were found to correspond to the insertion of PE70, PE93, and PE118, previously identified in patients' fibroblasts (Fig. 2A). Thus, this variant is responsible for the activation of the intronic pseudoexons, although some residual normal splicing is produced.

Variant c.243 + 3A>G was analyzed in a minigene with exon 4 and flanking intronic sequences cloned in the pSPL3 vector. As shown in Fig. 2B the c.243 + 3A>G variant causes exon 4 skipping, consistent with the predicted decrease in splice score of the mutant 5′ss and with the results obtained in fibroblasts. A faint band corresponding to the correctly spliced transcript is barely detectable, indicating that the normally spliced band observed in patient's fibroblasts (Fig. 1A) is probably mostly derived from the allele carrying the c.164-672C>T variant, which can be thus considered a “leaky” splicing variant.

The recognition of the pseudoexons by the splicing machinery may vary depending on the tissue analyzed, due to tissue specific differences in splice factors, as evidenced in several studies [12,49]. Therefore, we subsequently analyzed the effect on splicing of both variants c.243 + 3A>G and c.164-672C>T in SH-SY5Y neuroblastoma cells, given the relevant role of the *PTS* gene in the central nervous system. The results showed a similar splicing pattern for both variants (Fig. 2A, B).

### Pseudoexon exclusion with antisense oligonucleotides

**FIG. 3.**
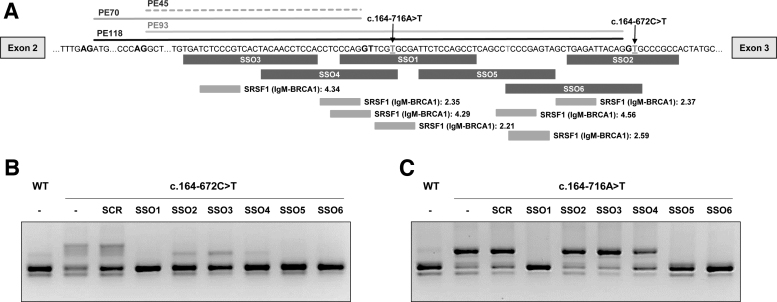
SSO treatment in minigenes. **(A)** Sequence of the intron 2 region showing the identified pseudoexons, the conserved GT and AG splice sites used, the location of the c.163–716A>T and c.164–672C>T variants, and the region targeted by the designed SSOs (*black boxes*). Binding sites for SRSF1 predicted by ESE finder are shown in light *grey boxes* along with their scores. **(B, C)** RT-PCR analysis in WT and mutant minigenes untransfected (−) or transfected with 50 nM of each SSO. SCR, scrambled SSO; SSO, splice switching antisense oligonucleotide.

Pathogenic pseudoexon inclusion resulting from deep intronic variants has the potential to be reverted using antisense therapy with SSOs. We designed six SSOs targeting the intronic region in which the different pseudoexons are located (Fig. 3A). A scrambled SSO was used as a nontarget control to discriminate between specific and unspecific effects. In this study we used full 2-OMe PS oligonucleotides, with SSO 1 having the wild-type sequence of the morpholino oligonucleotide (PTS-AMO3) used in a previous study to target PE45 and PE70 activated by variant c.164-716A>T (designated as c.164-712A>T in the previous study) [26]. SSO1 and SSO4 cover the 5′ss used by both these pseudoexons. SSO2 and SSO6 were designed for targeting the new 5′ss created by the novel variant c.164-672C>T. SSO3, which targets the TACAACCTC element previously suggested to be essential for PE45 inclusion [28]. SSO5 targets predicted potential binding sites for different SR splice factors.

Minigenes carrying variant c.164-672C>T were cotransfected with SSOs at 50 nM, and RT-PCR analysis showed that SSOs 1, 5, and 6 completely prevented pseudoexon inclusion (Fig. 3B). For cells transfected with SSOs 2, 3, and 4 a faint band corresponding in size to PE70, which is also detectable in the wild-type untreated minigene, remained after treatment (Fig. 3B).

The same SSOs were also used with the mutant minigene carrying the previously studied variant c.164-716A>T that results in the inclusion of PE45 and PE70 [26]. Again, SSOs 1, 5, and 6 completely prevented pseudoexon inclusion, and in this case, SSO 2, 3, and 4 had no apparent effect (Fig. 3C).

**FIG. 4.**
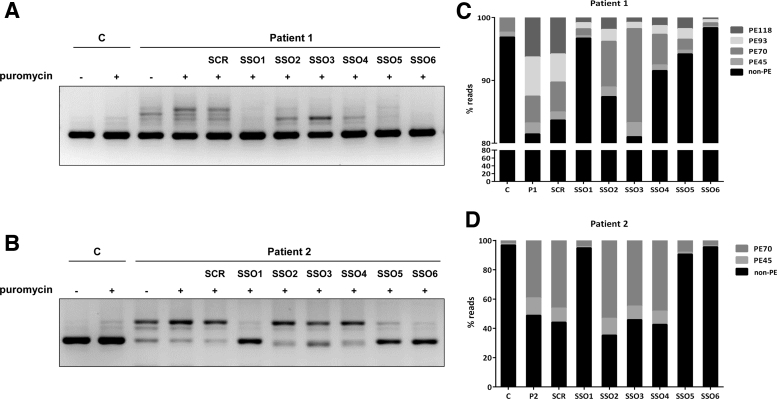
SSO treatment in patients' fibroblasts. RT-PCR analysis, using primers to amplify a fragment from exon 1 to exon 3, in control and patient 1 **(A)** or patient 2 **(B)** fibroblasts, transfected with 50 nM of each SSO. Puromycin was used as NMD inhibitor. **(C, D)** show the graphical representation of the quantification of each band corresponding to the transcripts without pseudoexons (non-PE) and to the insertion of each pseudoexon, expressed as percentage of reads obtained after deep sequencing the region in puromycin treated samples. NMD, nonsense mediated mRNA decay.

We next transfected patient 1 fibroblasts, treated with puromycin, to inhibit NMD, with the six SSOs. For the sake of clarity, in the RT-PCR analysis we used a reverse primer hybridizing to exon 3, to avoid detection of the exon skipping transcripts resulting from the allele with variant c.243 + 3A>G. The results were concordant with what we observe with minigenes, as SSO 1, 5, and 6 resulted in almost complete disappearance of the higher molecular weight bands, corresponding to pseudoexon inclusions, while SSOs 2, 3, and 4a band presumably corresponding to PE70 remained (Fig. 4A).

The effect of the six SSOs was also analyzed in patient 2 fibroblasts, heterozygous for c.164-716A>T that causes insertion of PE45 and PE70 [26]. Again, SSOs 1, 5, and 6 prevented pseudoexon inclusions and SSOs 2–4 showed no significant effect (Fig. 4B). In both patients 1 and 2 fibroblasts, a scrambled SSO had no effect on the splicing pattern.

Examining the hit SSOs we observe that SSO1 covers a sequence shared by all pseudoexons; however, SSOs 5 and 6 target sequences present only in the longer PE93 and PE118. To precisely define and quantify the effect of each of the SSOs on the inclusion of the different pseudoexons, we performed deep sequencing of the RT-PCR products from the transfected fibroblasts. The results are shown in Fig. 4C, D. In patient 1 cells, we observe similar levels (estimated by the number of reads) for PE70, PE93, and PE118, plus a low amount of PE45 (Fig. 4C). PE70 is predominant in patient 2 (Fig. 4D) and is also detectable in low amounts in control fibroblasts. SSO1 and 6 almost completely prevent all pseudoexon inclusion in both patients' fibroblasts, with SSO5 exhibiting a slightly lower effect. SSOs 2–4 prevalently hinder the inclusion of PE93 and PE118 and correspondingly have no effect in patient 2 cells (Fig. 4B, D).

### Analysis of splice factors binding to pseudoexon sequences

SSO1 hybridizes to a region included in all pseudoexons, and its effect could be the result of blocking the binding of splice factors critical for their aberrant inclusion. The positive effect of SSOs 5 and 6 that lie downstream of the 5′ss (for PE45 and PE70) or internal (PE93 and PE118) to the pseudoexons may also indicate the presence of splicing regulatory sites needed for pseudoexon recognition.

*In silico* analysis of the intronic sequences predict strong SRSF1 binding motifs in the region covered by hit SSOs 1, 5, and 6 (scores 4.3 and 4.6 according to ESEfinder) (Fig. 3A). To further investigate which splice factors bind to sequences targeted by SSOs 1, 5, and 6, we performed RNA affinity pull downs on biotinylated RNA oligonucleotides, containing the wild-type target sequences or with mutations that abolish the SRSF1 motifs (Supplementary Fig. S1). After incubation in HeLa cell nuclear extract, SR and hnRNPA1 proteins bound to each oligonucleotide were analyzed by SDS-PAGE and Western blotting. Using an anti-SR antibody there was no binding above background for SRSF2, SRSF3, SRSF4, SRSF5, or SRSF6 (data not shown). In addition, the pull downs with all RNA oligonucleotides showed little SRSF1 binding, although for PTS5 (antisense sequence to SSO5) we observe a stronger signal, which could be decreased by mutating the SRSF1 motif (Supplementary Fig. S1).

SPRi analysis of PTS5 shows binding of SRSF1 above background. When the potential SRSF1 binding site in PTS5 is disrupted by point mutations this binding is disrupted (Supplementary Fig. S2). SPRi analysis of PTS1 (sequence targeted by SSO1) and PTS6 (sequence targeted by SSO6) confirms the very low binding of SRSF1 observed in the pull-down experiments (Supplementary Fig. S2). Examination of the sequences using recombinant hnRNPA1 in the SPRi analysis indicated binding for all RNA oligonucleotides, confirming that they are all functional (Supplementary Fig. S2).

To provide additional evidence of SRSF1 binding to the targeted sequences, we examined publicly available SRSF1 CLIP datasets, including the K562 and HepG2 ENCODE eCLIP datasets [37], a PAR-CLIP dataset from HeLa cells [39], and a HITS-CLIP dataset from MDA-LM2 cells [40]. Normally, CLIP datasets are analyzed by considering only the uniquely aligned reads, but this overlooks real binding sites in duplicated sequences. Because the *PTS* PE is derived from the highly repeated *AluSq* sequence [28], reads would therefore normally be filtered away. We therefore aligned CLIP reads allowing for up to 100 multiple mapping locations to allow for mapping of reads to duplicated regions, including *AluY* sequence elements. This resulted in reads aligning at or on top of the potential SRSF1 binding sites in *PTS* (Supplementary Fig. S3), providing further evidence that these binding sites may be functional.

### PTPS protein recovery with SSO treatment

SSOs 1, 5, and 6 corrected the mis-splicing defect in patients 1 and 2 fibroblasts carrying deep-intronic variants activating pseudoexons in intron 2. To illustrate the therapeutic potential of the approach, we analyzed PTPS protein levels in cells treated with the different SSOs or a scrambled SSO. As using an NMD inhibitor may favor correctly spliced transcripts, interfering with the interpretation of the results, we analyzed cells treated or not with puromycin. Both fibroblast cell lines are compound heterozygous, carrying mis-splicing variants in both alleles, although some residual normal splicing can be detected (Fig. 1A).

**FIG. 5.**
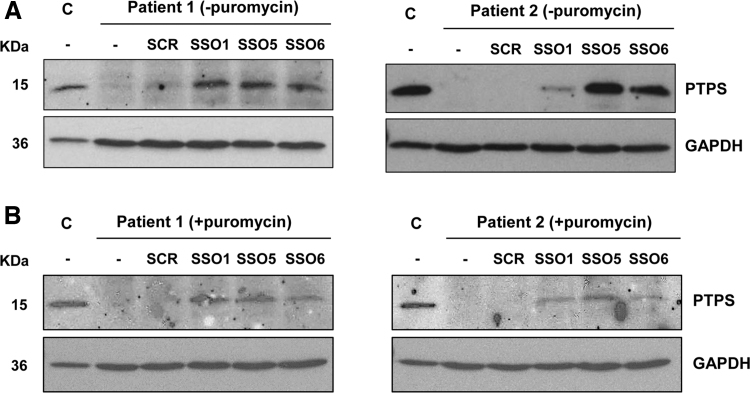
PTPS protein analysis in patients' fibroblasts. Western blot analysis of the PTPS protein in untransfected (−) or transfected fibroblasts from patients 1 and 2, untreated **(A)** or treated **(B)** with puromycin. GAPDH was used as loading control. C, control fibroblasts; PTPS, 6-pyruvoyltetrahydropterin synthase.

Western blot analysis revealed no detectable PTPS protein in untreated cells (Fig. 5). This confirms that in fibroblasts the resulting mis-spliced transcripts do not result in stable protein and that the protein translated from the residual normally spliced transcripts escapes detection limits with the antibody used. After transfection with the hit SSOs, PTPS protein is detected in both fibroblast cell lines, in cells treated or not with puromycin. Protein levels in patients' cells transfected with the different SSOs, estimated by densitometric analysis, ranged between 5% and 50%, relative to levels in control fibroblasts, depending on the SSO, treatment with or without puromycin and the cell line (Fig. 5).

## Discussion

Deep intronic variants that alter splicing are missed with standard exon-based diagnostic sequencing approaches. However, evidence has accumulated that such variants are an important class of disease-causing variants mainly due to the activation of pseudoexons [9,16]. Thus, it has been proposed that RNA analysis should be incorporated into the diagnostic workflow for patients with specific phenotypes and with no or incomplete genetic diagnosis [13]. In the present study routine clinical exome sequencing identified only one pathogenic *PTS* variant in patient 1 with a biochemical suspicion of PTPS deficiency. We subsequently used a candidate region approach, sequencing a deep-intronic region where previous studies had identified variants activating pseudoexons, followed by *PTS* transcript analysis in patients' fibroblasts. Interestingly, the analysis identified a novel intronic variant, c.164-672C>T, adding to the previously reported variants c.163 + 1695_1751del57 and c.164-716A>T in the same region [26]. The novel variant results in the exonization of previously described PE45 and PE70, plus two longer overlapping PE93 and PE118. These two are defined by the newly created 5′ss and the 3′ss of the previously reported pseudoexons.

Multiple pseudoexon inclusion due to a single intronic splice site coupled with different cryptic acceptor splice sites has already been described [50]. However, in this case, the novel variant also moderately increases the inclusion levels of upstream PE45 and PE70 compared to that observed in control fibroblasts, which could be due to the alleviation of some repressive mechanism, rather than to the occurrence of an activating event, as suggested in other cases [51]. In addition, some introns seem more prone to harbor disease-causing mutations than others, as observed in the ABCA4 gene in which the clustering in certain introns of deep-intronic variants causing Stargardt disease has been reported [52]. In the present study, the pseudoexons correspond to transposable *Alu* elements, which may lead to the creation of new exons in the genome or to aberrant disease-causing transcripts due to single point variants [8,21].

A very interesting aspect of our results is the fact that the new *PT*S c.164-672C>T variant creates a 5′ss identical to that used in the disease causing *Alu* derived *F8* pseudoexon that is activated by a deletion removing an inhibitory sequence in the 3′ss [24], and this 5′ss in *F8* is also activated when further strengthened by a point mutation altering the +3G>A also leading to disease [24,48]. Moreover, mutations in this sequence create the 5′ss most frequently used in naturally exonized *Alu*s [46,47]. The *PTS* PE118, the most prominent pseudoexon activated by the c.164-672C>T variant, is nearly identical to the *F8* PE, with exactly the same 3′ss and 5′ss. This underscores that similar mutations in the potential 5′ss, as that here reported in *PTS* and previously in *F8*, in the numerous *AluY* sequence elements present in introns in other genes are likely to be a frequent cause of disease causing pseudoexon activation.

Similarly, mutations in the same positions (205 and 206) in the *Alu* sequence as the 5′ss strengthened by the c.164–716A>T *PTS* variant have been reported to activate disease causing pseudoexons in *COL4A3* and *GUSB* genes [53,54], further supporting that these positions in the *Alu* sequences are high risk sites for disease causing pseudoexon activation.

Generally, most of the reported variants that activate pseudoexons either create or strengthen a preexisting suboptimal 5′ or 3′ ss [8,48], although in other pseudoexons, different types of activating variants have been identified [10,11]. In the *PTS* intron 2 region, one variant creates a novel 5′ss (c.164-672C>T), another strengthens a preexisting one (c.164-716A>T), and another brings closer a branch point sequence to the 3′ss (c.163 + 1695_1751del57) [26], revealing the spectrum of rare deleterious variants activating pseudoexons in this region.

Following the identification of the aberrant insertions, SSOs were designed with the aim of restoring normal splicing by forcing the skipping of the pathogenic pseudoexons. This strategy has been validated in minigene assays and in cultured patient-derived cells in a wide range of disorders and has efficiently shown the rescue of these loss-of-function variants [8,22,30]. Our results showed that both in minigenes and in the endogenous gene context using patients' fibroblasts, SSOs 1 and 6 (and to a slightly lesser extent, SSO5) were most effective for all *PTS* intron 2 pseudoexons, providing support for the potential of using a single SSO to treat patients with different pathogenic variants in the region. The relative amounts of each pseudoexon in the final transcript profile in patients' fibroblasts were measured using deep sequencing. This approach represents an efficient way to precisely quantify the efficacy of SSO-mediated splice correction for adjacent or overlapping pseudoexons.

The presence of SRSF1 motifs in the pseudoexon sequences indicated a possible mechanism of action of the hit SSOs, whereby they would block binding of this splice factor to the predicted sites, thus precluding pseudoexon inclusion, as previously described [55,56]. Taken together, our results using RNA pull downs and SPRi and analysis of CLIP data suggest that SSO5 could indeed function by blocking access to an SRSF1 ESE (CTCCCGAGTA) with two predicted overlapping binding sites (CTCCCGA—Score 4.56) and (CCGAGTA—Score 2.59) (Fig. 3A). When these sites are abolished by point mutations, binding decreases (Supplementary Figs. S1, S2). SSO6 covers most of the high score (4.56) SRSF1 motif; thus, it is plausible that it will block SRSF1 binding and cause exon skipping, although as the entire motif is not present, experimentally we observe low SRSF1 binding to the target sequence (Supplementary Figs. S1, S2).

SSO1 blocks the 5′ss of PE45 and PE70, which may contribute to the exclusion of these pseudoexons. In addition, it is known that secondary RNA structure can affect pre-mRNA splicing, regulating accessibility of cis-acting or trans-acting elements and the spatial distance in between [57]. In this case, SSO binding may also influence local RNA structure favoring pseudoexon exclusion.

SSO6 targets a region that encompasses the mutated nucleotide at position c.164-672C>T and, therefore, contains a mismatch toward the sequence in patient 2. Contrary to what has been reported in some studies [52,58], this single mismatch between the SSO and its target sequence apparently does not affect its splicing modulation capacity. Similarly, SSO1 with the wild-type sequence functions in patient 2 samples, despite the presence of a mismatch at the site of the c.164-716A>T variant. Of note, it can be envisaged that these same hit SSOs can be used for pseudoexon activating variants in similar *Alu*-derived activated pseudoexons, as the above mentioned are nearly identical in the *F8* gene. This implies for the first time, to our knowledge, that one single SSO could potentially be used for treating patients with different diseases.

Comparing the results obtained in a previous study [26], in which the phosphorodiamidate morpholino oligonucleotide (PMO) chemistry was used at concentrations 20–30 μM, we observe similar splicing correction (pseudoexon exclusion) in patient 2 fibroblasts transfected with SSO1, same sequence as PTS-AMO3, but with 2-OMe-PS chemistry (see Fig. 3 in [26], compared to Fig. 4, this work). Both chemistries have proven to be effective *in vitro*, although *in vivo,* the neutral charge of PMOs improves tolerability as it results in lower binding to plasma proteins. Currently, 3 SSOs with PMO chemistry have received FDA approval for the treatment of Duchenne muscular dystrophy [59].

Protein analysis confirmed the recovery of PTPS protein levels after transfection of patient-derived fibroblasts with hit SSOs 1, 5, and 6. Both patients are compound heterozygous, carrying the pseudoexon activating variant in one allele. Although PTPS enzymatic activity could not be determined in this study, the levels of protein achieved here have been previously shown to correlate with a therapeutically relevant increase in enzymatic activity [26].

It must be noted that for rare pathogenic variants, occurring only in one or two families, developing a personalized genetic therapy represents a challenge, although the proof of concept of the feasibility of such an approach for a single patient with a deep intronic variant was recently reported [25] and more n-of-1 RNA therapy trials are being developed in academic or pharmaceutical settings [60,61]. Indeed, the recently founded n-Lorem Foundation aims to discover and develop antisense oligonucleotides for patients with unique mutations and to provide them for free as treatment for life [62]. Another important hurdle in preclinical development for SSOs targeting pseudoexons is the difficulty in generating adequate animal models, as intronic sequences are not conserved among species. However, with the advent of CRISPR/Cas gene editing technologies it is now feasible to easily generate humanized mouse models carrying the desired intronic pseudoexon sequences, although reproducibility of the splicing defect may depend on the whole genomic context and thus should be first confirmed *in vitro* (e.g., using minigenes or a similarly edited mouse cell line).

PE70 that corresponds to the “natural” predicted *Alu* sequence [28] is detectable in low abundance in the wild type context, as evidenced in minigene assays (Figs. 2, 3) and in control fibroblasts (Fig. 4). Residual pseudoexon inclusion leading to a nonfunctional transcript in control individuals has been detected for several genes involved in disease [63,64]. It was initially considered an undesired unproductive splicing event. However, evidence has accumulated indicating that it is actually a well-represented mechanism of gene expression regulation [65]. During specific dynamic cellular processes, genes may be subjected to alternative splicing, resulting in such nonfunctional transcripts rapidly regulating final mRNA and protein levels [66]. Recently, this knowledge was exploited applying antisense technology to prevent nonproductive pseudoexon inclusion events, resulting in an increase in functional mRNA and protein levels in monogenic diseases characterized by haplo-insufficiency [67,68]. The authors detected by bioinformatic analysis more than 1,000 nonproductive splicing events in disease associated genes and using this approach, termed TANGO (targeted augmentation of nuclear gene output), confirmed an increase in productive mRNA and protein for different genes *in vitro* and *in vivo* [67].

The antisense approach targeting naturally occurring pseudoexon inclusion events could be applied to the *PTS* gene in the case of missense variants retaining residual activity. Over 100 variants have been reported in the *PTS* gene associated with PTPS deficiency, mostly missense variants [69], many of them affecting protein structure and stability [70–72]. It is also known that the milder peripheral forms of PTPS deficiency are associated to partial activity [73]. Future experiments to investigate the feasibility of the TANGO approach can be considered using fibroblasts derived from patients with such genotypes.

The present work adds to the current knowledge of the relatively high prevalence of variant induced pseudoexon inclusion as a disease mechanism in inherited metabolic disorders [74]. These variants are promising candidates for personalized therapies using SSOs, which, along with other RNA-therapies, have registered a boost in the last few years, with more than a dozen drugs approved for clinical use and many others currently in different stages of development. It can be anticipated that this type of drugs may shortly be in use for one or very few patients (personalized medicine) or to treat common diseases, once delivery and toxicity issues are solved [19].

## Supplementary Material

Supplemental data

Supplemental data

Supplemental data

Supplemental data

Supplemental data
