# Supplementary material for: Antisense Oligonucleotide Rescue of Deep-Intronic Variants Activating Pseudoexons in the 6-Pyruvoyl-Tetrahydropterin Synthase Gene
Source: Nucleic Acid Ther. 2022 Oct 14;32(5):378–90. doi: 10.1089/nat.2021.0066 (PMC9595628; doi:10.1089/nat.2021.0066)
Supplement: Supplemental data [file Supp_TableS1.pdf]

Table S1. SSO length and sequence.

SSOs	Length (bp)	Sequence 5’-3’
SSO Scrambled	20	CTCAATATGCTACTGCCATG
SSO1	25	AGGCTGGAGAATCGCTCGAACCTGG
SSO2	20	GTGGCGGGCACCTGTAATCT
SSO3	25	TGGAGGTTGTAGTGACGGGAGATCA
SSO4	25	TCGAACCTGGGAGGTGGAGGTTGTA
SSO5	25	TACTCGGGAGGCTGAGGCTGGAGAA
SSO6	25	CACCTGTAATCTCAGCTACTCGGGA
