# Supplementary material for: Antisense Oligonucleotide Rescue of Deep-Intronic Variants Activating Pseudoexons in the 6-Pyruvoyl-Tetrahydropterin Synthase Gene
Source: Nucleic Acid Ther. 2022 Oct 14;32(5):378–90. doi: 10.1089/nat.2021.0066 (PMC9595628; doi:10.1089/nat.2021.0066)
Supplement: Supplemental data [file Supp_FigS1.pdf]

A)

PTS1	CCAGGUUCGAGCGAUUCUCCAGCCU-biotin
PTS1 Mut1	CCAGGUUCGUGCGAUUCUCCAGCCU-biotin
PTS1 Mut2	CCAGGUUCUAGCUAUUCUCCAGCCU-biotin
PTS1 Rev	AGGCUGGAGAAUCGCUCGAACCUGG-biotin
PTS5	UUCUCCAGCCUCAGCCUCCCGAGUA-biotin
PTS5 Mut	UUCUCCAGCCUCAGCCUCUUGAGUA-biotin
PTS5 Rev	UACUCGGGAGGCUGAGGCUGGAGAA-biotin
PTS6	UCCCGAGUAGCUGAGAUUACAGGUG-biotin
PTS6 Mut	UCUUGUGUAGCUUAUAUUUCUGGUG-biotin
PTS6 Rev	CACCUGUAAUCUCAGCUACUCGGGA-biotin

B)

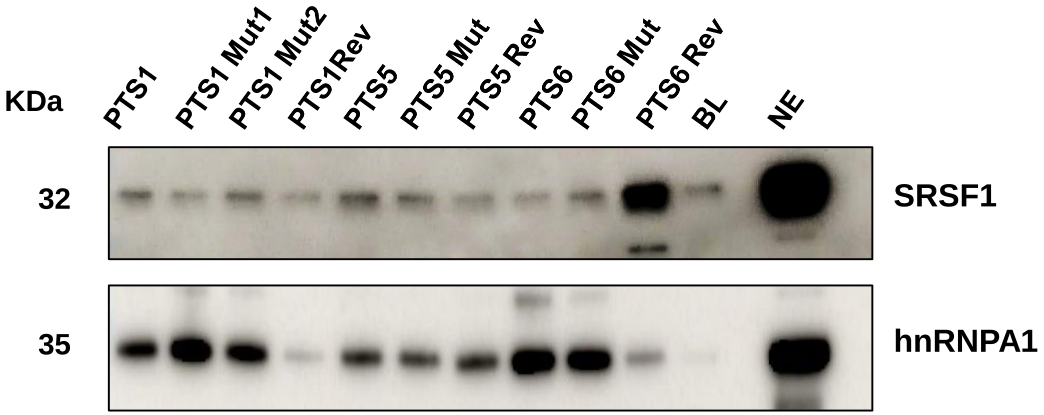

Figure S1. RNA affinity purification. A) Sequences of biotin-conjugated RNA oligonucleotides covering the three SSO1, 5 and 6 binding sites of *PTS* intron 2. Mutations disrupting the SRSF1 binding motifs are shown in red. B) Western blot of SRSF1 and hnRNPA1 proteins purified by RNA-affinity chromatography. Results are representative for two experiments. BL and NE indicate control lanes without RNA oligonucleotides or with nuclear extract alone, respectively.
