# Supplementary material for: Antisense Oligonucleotide Rescue of Deep-Intronic Variants Activating Pseudoexons in the 6-Pyruvoyl-Tetrahydropterin Synthase Gene
Source: Nucleic Acid Ther. 2022 Oct 14;32(5):378–90. doi: 10.1089/nat.2021.0066 (PMC9595628; doi:10.1089/nat.2021.0066)
Supplement: Supplemental data [file Supp_FigS2.pdf]

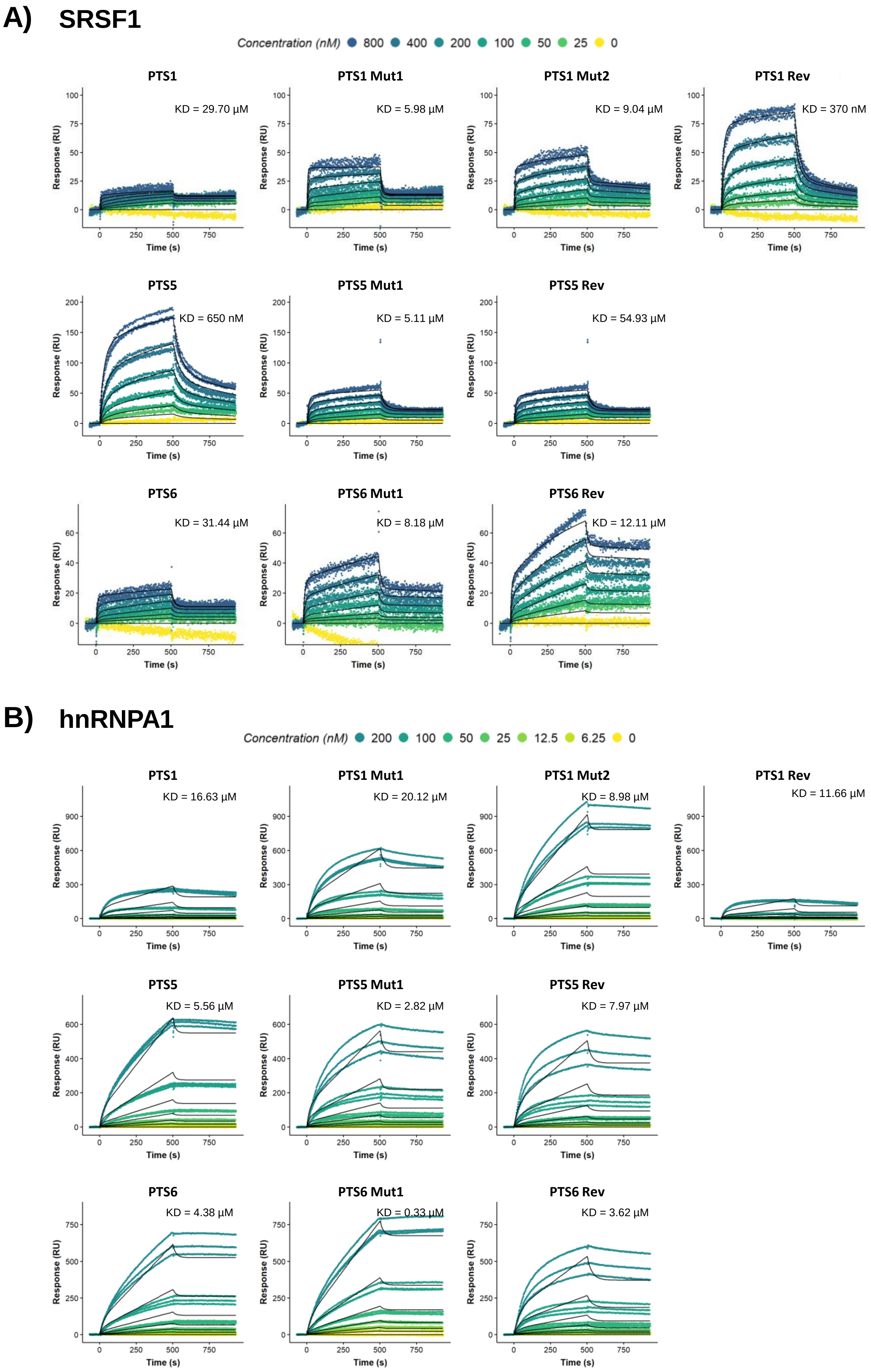

Figure S2. Surface plasmon resonance imaging (SPRi) of SRSF1 and hnRNP A1 recombinant proteins. SRSF1 and hnRNP A1 were injected in increasing 2-fold concentrations from 25 to 800 nM or 6.25 to 200 nM, respectively. Measurements were fitted to a bimodal 1:1. For hnRNPA1 a biphasic 1:2 model was used in ClampXP.
