# Supplementary material for: Antisense Oligonucleotide Rescue of Deep-Intronic Variants Activating Pseudoexons in the 6-Pyruvoyl-Tetrahydropterin Synthase Gene
Source: Nucleic Acid Ther. 2022 Oct 14;32(5):378–90. doi: 10.1089/nat.2021.0066 (PMC9595628; doi:10.1089/nat.2021.0066)
Supplement: Supplemental data [file Supp_Data.pdf]

SUPPLEMENTARY METHODS

Processing of eCLIP datasets. Raw SRSF1 eCLIP reads [1] were downloaded from the ENCODE website and trimmed twice using cutadapt v1.15 [2]. Details of the run parameters are described in Supplementary methods. for the first round of trimming:

cutadapt -f fastq --match-read-wildcards --times 1 -e 0.1 -O 1 --quality-cutoff 6 -m 18 -a NNNNNAGATCGGAAGAGCACACGTCTGAACTCCAGTCAC -g CTTCCGATCTACAAGTT -g CTTCCGATCTTGGTCCT -A AACTTGTAGATCGGA -A AGGACCAAGATCGGA -A ACTTGTAGATCGGAA -A GGACCAAGATCGGAA -A CTTGTAGATCGGAAG -A GACCAAGATCGGAAG -A TTGTAGATCGGAAGA -A ACCAAGATCGGAAGA -A TGTAGATCGGAAGAG -A CCAAGATCGGAAGAG -A GTAGATCGGAAGAGC -A CAAGATCGGAAGAGC -A TAGATCGGAAGAGCG -A AAGATCGGAAGAGCG -A AGATCGGAAGAGCGT -A GATCGGAAGAGCGTC -A ATCGGAAGAGCGTCG -A TCGGAAGAGCGTCGT -A CGGAAGAGCGTCGTG -A GGAAGAGCGTCGTGT -o reads.R1.trimmed.fastq.gz -p reads.R2.trimmed.fastq.gz reads.R1.fastq.gz reads.R2.fastq.gz

The second round of trimming was run with the following parameters:

cutadapt -f fastq --match-read-wildcards --times 1 -e 0.1 -O 5 --quality-cutoff 6 -m 18 -A AACTTGTAGATCGGA -A AGGACCAAGATCGGA -A ACTTGTAGATCGGAA -A GGACCAAGATCGGAA -A CTTGTAGATCGGAAG -A GACCAAGATCGGAAG -A TTGTAGATCGGAAGA -A ACCAAGATCGGAAGA -A TGTAGATCGGAAGAG -A CCAAGATCGGAAGAG -A GTAGATCGGAAGAGC -A CAAGATCGGAAGAGC -A TAGATCGGAAGAGCG -A AAGATCGGAAGAGCG -A AGATCGGAAGAGCGT -A GATCGGAAGAGCGTC -A ATCGGAAGAGCGTCG -A TCGGAAGAGCGTCGT -A

```
CGGAAGAGCGTCGTG -A GGAAGAGCGTCGTGT -o reads.R1.trimmed2.fastq.gz -p reads.R2.trimmed2.fastq.gz reads.R1.trimmed.fastq.gz reads.R2.trimmed.fastq.gz
```

For each read file, we then prepared the barcode for PCR-duplication detection with UMI tools by appending it to the read ID:

```
awk -v l=10 'BEGIN{OFS=FS=" "} substr($1, 1, 1) == "@" {print "@" substr($1, (l+3), 500) "_" substr($1, 2, l) " " $2 }; substr($1, 1, 1) != "@" {print}; ' <(zcat reads.R1.trimmed2.fastq.gz)  | gzip > reads.R1.trimmed2.bc.fastq.gz
```

Subsequently, reads were mapped with STAR v2.7.8a [3] to the human genome using GENCODE v39 hg38 annotations allowing for up to 100 mapping locations with the following run parameters:

```
star --outSAMtype BAM SortedByCoordinate --runThreadN 40 --genomeDir star_index --readFilesIn reads.R1.trimmed2.bc.fastq.gz reads.R2.trimmed2.bc.fastq.gz --readFilesCommand zcat --outFilterType BySJout --outFilterMultimapNmax 100 --alignSJoverhangMin 8 --alignSJDBoverhangMin 1 --outFilterMismatchNmax 999 --outFilterMismatchNoverLmax 0.04 --scoreDelOpen -1 --alignIntronMin 20 --alignIntronMax 1000000 --alignMatesGapMax 1000000 --alignEndsType EndToEnd --outMultimapperOrder Random --winAnchorMultimapNmax 100
```

Using UMI-tools [4], PCR duplicates were removed with the following command:

```
umi_tools dedup -I Aligned.sortedByCoord.out.bam --paired -S Aligned.sortedByCoord.rm-pcr-dup.bam
```

Subsequently, only read 2 was retained, to mark sites of protein binding, using samtools [5]:

```
samtools view -hb -f 130 Aligned.sortedByCoord.rm-pcr-dup.bam -o Aligned.sortedByCoord.rm-pcr-dup.R2.bam
```

Finally, we merged the two replicates for each cell type using samtools merge.

Processing of PAR-CLIP dataset: Raw PAR-CLIP reads were downloaded from the GEO repository using the accession number GSE71095 [6] and trimmed using cutadapt with the following run parameters:

cutadapt -f fastq -u 5 -q 15 -m 20 -a TGGAATTCTCGGGTGCCAAGG -o reads.trimmed.fastq.gz reads.fastq.gz

We then mapped the reads to the human genome using GENCODE v39 hg38 annotations allowing for up to 100 mapping locations with the following run parameters:

star --outSAMtype BAM SortedByCoordinate --runThreadN 40 --genomeDir star_index --readFilesIn reads.trimmed.fastq.gz --readFilesCommand zcat --outFilterType BySJout --outFilterMultimapNmax 100 --alignSJoverhangMin 8 --alignSJDBoverhangMin 1 --outFilterMismatchNmax 999 --outFilterMismatchNoverLmax 0.04 --scoreDelOpen -1 --alignIntronMin 20 --alignIntronMax 1000000 --alignMatesGapMax 1000000 --alignEndsType Local --outMultimapperOrder Random –winAnchorMultimapNmax 100

Subsequently, we removed PCR duplicates using samtools rmdup.

Processing of HITS-CLIP dataset: Raw HITS-CLIP reads were downloaded from the GEO repository using the accession numbers GSE131745 [7] and trimmed using cutadapt with the following run parameters:

cutadapt -f fastq -u 5 -q 15 -m 20 -a GTGTCAGTCACTTCCAGCGG -o reads.trimmed.fastq.gz reads.fastq.gz

We then mapped the reads to the human genome using GENCODE v39 hg38 annotations allowing for up to 100 mapping locations with the following run parameters:

star --outSAMtype BAM SortedByCoordinate --runThreadN 40 --genomeDir star_index --readFilesIn reads.trimmed.fastq.gz --readFilesCommand zcat --outFilterType BySJout --outFilterMultimapNmax 100 --alignSJoverhangMin 8 --alignSJDBoverhangMin 1 --outFilterMismatchNmax 999 --outFilterMismatchNoverLmax 0.04 --scoreDelOpen -1 --

alignIntronMin 20 --alignIntronMax 1000000 --alignMatesGapMax 1000000 --alignEndsType Local --outMultimapperOrder Random –winAnchorMultimapNmax 100

Subsequently, we removed PCR duplicates using samtools rmdup and merged the two replicate experiments using samtools merge.

References

1. Van Nostrand, E.L. et al. (2016) Robust transcriptome-wide discovery of RNA-binding protein binding sites with enhanced CLIP (eCLIP). Nat Methods 13 (6), 508-14.
2. Martin, M. (2011) Cutadapt removes adapter sequences from high-throughput sequencing reads. EMBnet.Journal 17 (1), 10-12.
3. Dobin, A. et al. (2013) STAR: ultrafast universal RNA-seq aligner. Bioinformatics 29 (1), 15-21.
4. Smith, T. et al. (2017) UMI-tools: modeling sequencing errors in Unique Molecular Identifiers to improve quantification accuracy. Genome Res 27 (3), 491-499.
5. Li, H. et al. (2009) The Sequence Alignment/Map format and SAMtools. Bioinformatics 25 (16), 2078-9.
6. Xiao, W. et al. (2016) Nuclear m(6)A Reader YTHDC1 Regulates mRNA Splicing. Mol Cell 61 (4), 507-519.
7. Fish, L. et al. (2019) Nuclear TARBP2 Drives Oncogenic Dysregulation of RNA Splicing and Decay. Mol Cell 75 (5), 967-981 e9.
