# Supplementary material for: Antisense Oligonucleotide Rescue of Deep-Intronic Variants Activating Pseudoexons in the 6-Pyruvoyl-Tetrahydropterin Synthase Gene
Source: Nucleic Acid Ther. 2022 Oct 14;32(5):378–90. doi: 10.1089/nat.2021.0066 (PMC9595628; doi:10.1089/nat.2021.0066)
Supplement: Supplemental data [file Supp_FigS3.pdf]

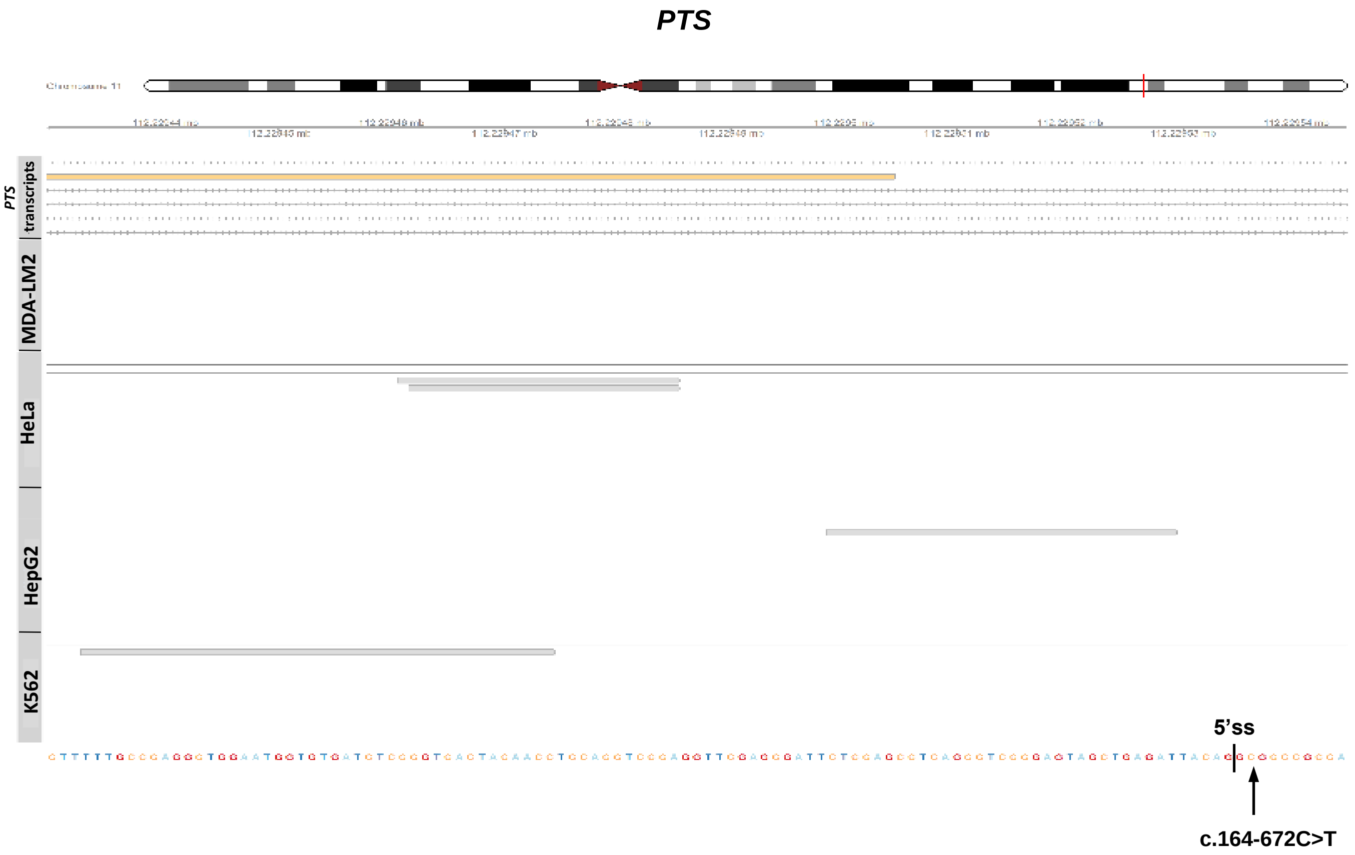

Figure S3. SRSF1 CLIP reads mapped to the pseudoexon region within the *PTS gene*. The cell types of the originating sample are indicated on the left side. The position of the novel 5'ss of the pseudoexon in PTS is indicated by a black bar on the sequence track at the bottom, and the c.164-672C>T variant by a black arrow.
